# THE ASPREE STUDY: DISABILITY-FREE SURVIVAL, UPDATED RESULTS, SUB-STUDIES, AND IMPLICATIONS FOR ASPIRIN USE

**DOI:** 10.1093/geroni/igz038.2356

**Published:** 2019-11-08

**Authors:** Anne M Murray, John McNeil, Basil Eldadah

**Affiliations:** 1 Berman Center for Outcomes and Clinical Research, Minneapolis, Minnesota, United States; 2 Department of Epidemiology & Preventive Medicine, Monash University, Melbourne VIC, Australia, Melbourne VIC, Australia, Australia; 3 National Institute on Aging, NIH, Bethesda, Maryland, United States

## Abstract

The NIA/NCI ASPREE (ASPirin in Reducing Events in the Elderly) Study was a landmark RCT of 19,114 healthy adults aged 70 (whites) and 65 (US minorities) in Australia and the US that demonstrated lack of effect of low dose aspirin (LDA:100 mg/d) on the novel primary end point of Disability- Free Survival (life free of disability and dementia) over a mean treatment of 4.7 years. Surprisingly, LDA was associated with a trend toward increased all cause mortality, driven by cancer deaths (results published NEJM September 2018). After the LDA intervention was halted in June 2017, ASPREE was extended as an observational cohort follow-on study, ASPREE-XT, to measure potential delayed LDA effects on ASPREE outcomes. The ASPREE study primary results will be summarized, and the rationale for and performance of the novel DFS geriatric outcome discussed. New results of the analysis of dementia as a secondary outcome will also be presented (both for overall dementia and Alzheimer’s disease). We will also examine the unexpected increased all-cause mortality attributed to cancer deaths, despite no significant difference between groups for all incident cancer, and effects of LDA on incident metastatic disease. The important implications of the ASPREE results for prescribing LDA for primary prevention in health elderly will be discussed, and the ASPREE-XT study design and progress described. Lastly, the breadth of the ASPREE sub-studies including the Biobank, Brain Imaging studies and Genomics, and opportunities to access the rich ASPREE data and collaborate with ASPREE investigators will be reviewed.

